# Complete Coding Genome Sequence of Putative Novel Bluetongue Virus Serotype 27

**DOI:** 10.1128/genomeA.00016-15

**Published:** 2015-03-12

**Authors:** Maria Jenckel, Emmanuel Bréard, Claudia Schulz, Corinne Sailleau, Cyril Viarouge, Bernd Hoffmann, Dirk Höper, Martin Beer, Stéphan Zientara

**Affiliations:** a Institute of Diagnostic Virology, Friedrich-Loeffler-Institut, Greifswald-Insel Riems, Germany; b ANSES Alfort, UMR 1161 ANSES/INRA/ENVA, Maisons-Alfort, France

## Abstract

We announce the complete coding genome sequence of a novel bluetongue virus (BTV) serotype (BTV-n = putative BTV-27) detected in goats in Corsica, France, in 2014. Sequence analysis confirmed the closest relationship between sequences of the novel BTV serotype and BTV-25 and BTV-26, recently discovered in Switzerland and Kuwait, respectively.

## GENOME ANNOUNCEMENT

Bluetongue is a vector-borne, infectious, notifiable disease of ruminants caused by bluetongue virus (BTV), an orbivirus within the *Reoviridae* family. The BTV genome consists of 10 segments of double-stranded RNA. A total of 26 different serotypes have been recorded (1–4).

In early 2014, a novel bluetongue virus (putative BTV-27) was coincidentally detected in clinically healthy goats during a BTV vaccination and monitoring program in Corsica, France (5). The origin of this virus remains unknown. The novel BTV isolate obtained during the field study was passaged on BSR cells (5). Sequence analysis of the serotype-specific segment (Seg)-2 showed the closest relationship to BTV-25 (73% nucleotides [nt], 75% amino acids [aa]) and BTV-26 (65% nt, 60% aa), which were recently detected in goats in Switzerland and Kuwait, respectively (2, 4, 5). A new serotype was suggested, because differences at the nucleotide and amino acid levels, respectively, of Seg-2 exceeded the overall interserotype variations reported previously (5, 6).

For a more comprehensive characterization, the coding regions of the remaining nine segments were sequenced. RNA was extracted using TRIzol LS reagent (LifeTechnologies, Darmstadt, Germany) and an RNeasy minikit (Qiagen, Hilden, Germany) with on-column DNase digestion according to the manufacturer’s recommendations. RNA was converted into double-stranded DNA using a cDNA synthesis system (Roche, Mannheim, Germany). Library preparation was conducted as described (7). Sequencing was carried out with an Illumina MiSeq Instrument using the MiSeq reagent kit version 3 (Illumina, San Diego, CA, USA).

Sequence data were assembled using the Genome Sequencer version 2.6 software suite (Roche) and BTV-related contigs were identified with BLASTn (BLASTn 2/2/26+; http://blast.ncbi.nlm.nih.gov/Blast.cgi). Potential assembly errors were eliminated by reference mapping along the identified contigs.

The highest identities of the full-coding genome of putative BTV-27 were found with BTV-25 and BTV-26. Of the 10 segments, six (Seg-1 to Seg-4, Seg-6, Seg-8) showed the highest similarities with BTV-25 at the nucleotide level, seven (additionally Seg-9) at the amino acid level: 73% nt (Seg-2) to 86% nt (Seg-1) and 67% aa (Seg-9) to 95% aa (Seg-3). The remaining segments were most closely related to BTV-26: 76% nt (Seg-9) to 84% nt (Seg-10) and 88% aa (Seg-5) to 97% aa (Seg-7).

Interestingly, our results suggest that the nonstructural protein 2 encoded by Seg-8 might start nine codons before the start codon known from other BTV genotypes. A missing nucleotide within the 5′ UTR leads to a frameshift and consequently enables an early start codon. All remaining encoded proteins fitted the size known for all other BTV isolates available in GenBank.

This is the first report of the complete coding genome of a recently discovered Corsican BTV isolate, putative new serotype 27. The sequence will facilitate the analyses of origin and introduction route of this virus to Corsica. Furthermore, it will give additional information about the genotype and serotype of this isolate. Animal trials required to evaluate the pathogenesis after inoculation are in progress.

### Nucleotide sequence accession numbers.

The complete coding genome sequences of the novel putative BTV-27 have been deposited in DDBJ/ENA/GenBank under the accession numbers LN713671 through LN713679 and KM200718. The version described in this paper is the first version.
